# Evaporative Morphology Tuning of Conducting Polymer Films Under Controlled Vacuum Conditions

**DOI:** 10.1002/advs.202408790

**Published:** 2024-12-23

**Authors:** Seongju Kim, Byung Mook Weon, Dong Jin Kang, Sungjune Jung

**Affiliations:** ^1^ Department of Materials Science and Engineering Pohang University of Science and Technology (POSTECH) 77 Cheongam‐Ro, Nam‐gu Pohang 37673 Republic of Korea; ^2^ Soft Matter Physics Laboratory School of Advanced Materials Science and Engineering SKKU Advanced Institute of Nanotechnology (SAINT) Sungkyunkwan University Suwon 16419 Republic of Korea; ^3^ Display Research Center Samsung Display Co., Ltd 1 Samsung‐ro, Giheung‐gu Yongin‐city Gyeonggi‐Do 17113 Republic of Korea

**Keywords:** coffee‐ring effect, diffusion, evaporation mode, PEDOT:PSS, sessile drop

## Abstract

The evaporation of drops on solid surfaces is a ubiquitous natural phenomenon, and their dynamics play a pivotal role in many biological, environmental, and industrial processes. However, the complexity of the underlying mechanisms has largely confined previous studies to liquid drop evaporation under atmospheric conditions. In this study, the first comprehensive investigation of the evaporation dynamics of conducting polymer‐containing drops under controlled vacuum environments is presented. Utilizing high‐speed imaging of a drop within a vacuum chamber, it is observed that the evaporation of a sessile drop under vacuum conditions unfolds through four distinct stages: Constant Contact Radius (CCR), Constant Contact Angle (CCA), Increasing Contact Angle (ICA), and Stick and Slip (S&S) modes. The detailed analysis of the force balance reveals that the depinning dynamics of the contact line, significantly driven by vacuum‐induced forces, are the primary factor distinguishing these evaporation modes. A modified diffusion‐limited model specifically tailored for vacuum conditions is further developed, which closely aligns with the experimental data on volume reduction over time. Importantly, the study demonstrates that by carefully adjusting the vacuum level, it is possible to precisely manipulate the final film morphology, with uniform deposition achieved at an optimal pressure of 20 kPa. This research introduces a novel approach for controlling drop evaporation dynamics in vacuum, with potential applications in advanced manufacturing processes.

## Graphical abstract

The evaporation of the sessile drop under vacuum conditions progresses through four distinct modes: Constant Contact Radius (CCR), Constant Contact Angle (CCA), Increasing Contact Angle (ICA), and Stick and Slip (S&S) modes. The evolution of evaporation modes under varying ambient pressures significantly influences the resulting deposition patterns of conducting polymers on the substrate.

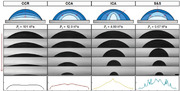

## 1 Introduction

Conducting polymers are promising materials for a wide range of applications, including flexible semiconductors and sensors,^[^ ^1^, ^2^, ^3^, ^4^ ^]^ next‐generation displays,^[^ ^5^, ^6^, ^7^ ^]^ energy devices^[^ ^8^, ^9^, ^10^, ^11^ ^]^ and bioelectronics.^[^ ^12^, ^13^, ^14^ ^]^ Solution processes such as printing and coating are commonly used to fabricate conducting polymer structures and devices. In these processes, evaporation is the final stage of a droplet and has a significant impact on the performance of the resulting products. Therefore, a comprehensive understanding of the evaporation dynamics of a small sessile drop on a solid substrate is essential. In manufacturing, reducing evaporation time is critical to maximizing productivity while ensuring the desired morphology of functional materials after drying.^[^ ^15^, ^16^, ^17^ ^]^ Consequently, a simple, controllable, and cost‐effective deposition technique for conducting polymers is essential to consistently produce a uniform film morphology.

The evaporation process is governed by the diffusion of vapor in its ambient environment.^[^ ^18^, ^19^ ^]^ Two pure evaporation modes can emerge for a small drop on a smooth surface, determined by pinning or depinning of contact lines on the surface.^[^ ^20^ ^]^ The constant contact radius (CCR) mode that commonly occurs on a wettable substrate leads to the most common outcomes of this process, known as the “coffee‐ring effect.”^[^ ^21^ ^]^ On the other hand, the constant contact angle (CCA) mode is predominant on surfaces with a weak interaction between the drop and the substrate. The evaporation of a sessile drop can exhibit a mixed evaporation mode, where the contact radius and angle change simultaneously during evaporation. Strategies to modify the evaporation mode have been extensively investigated for applications requiring uniform thin film morphology to control the deposition patterns.^[^ ^22^, ^23^ ^]^ These strategies range from altering the treatment of substrate properties^[^ ^24^, ^25^, ^26^, ^27^ ^]^ and liquid surface temperature,^[^ ^28^ ^]^ manipulating the evaporation rate by heating the substrate,^[^ ^29^, ^30^, ^31^, ^32^ ^]^ to tuning particle characteristics,^[^ ^33^, ^34^, ^35^ ^]^ all performed in atmospheric conditions. Despite these technological advances, the manufacturing sector faces the critical requirement of precisely controlling the evaporating mode. This control must be achieved without compromising the quality of the final products and concurrently should contribute to reducing evaporation time, thereby enhancing manufacturability.

This study investigates the evaporation dynamics of a sessile drop containing the conducting polymer under various pressure conditions, ranging from 101 kPa (atmospheric pressures: 1ATM) to 0.67 kPa. Utilizing a custom‐built vacuum chamber integrated with a high‐speed imaging system, we meticulously visualize and analyze the transitions through distinct evaporation modes as a function of the pressure levels. We propose a modified Young‐Laplace equation to account for the balance of interfacial stresses under vacuum conditions. Additionally, the implementation of a modified diffusion‐limited evaporation model that incorporates an ambient pressure factor effectively captures the evolution of drop volume under these varied conditions. Significantly, the changes in evaporation modes under different vacuum pressures have a profound impact on the deposition patterns of the conductive polymer PEDOT:PSS. This effect is particularly notable in the transition from Constant Contact Radius (CCR) to Constant Contact Angle (CCA) modes, which promotes uniform film formation at a specific critical pressure level. This research not only reveals, for the first time, the detailed dynamics of drop evaporation in a vacuum environment, but also demonstrates its potential for precise control over deposition processes, offering significant advancements in modern manufacturing applications.

## 2 Results and Discussion

**Figure 1:**
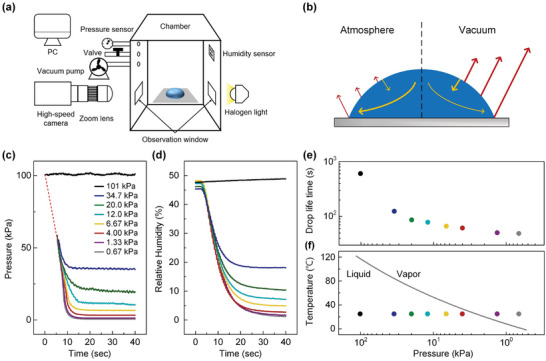
a) Schematic drawing of atmospheric evaporation and vacuum evaporation. b) The schematic diagram of atmosphere and vacuum evaporation. c,d) Temporal evolution of chamber pressure and relative humidity at different final pressure settings. e) The lifetime of an evaporation drop under different pressures. f) The dependence of the water boiling point on pressure.

The evaporation of drops is primarily governed by the diffusion of vapor in the surrounding air. In this research, we explore how varying the vacuum levels in the ambient environment affects the evaporation mode of a sessile drop and its consequent impact on the final morphology of the dried film. An aqueous solution of poly(3,4‐ethylenedioxythiophene):poly(styrene sulfonate) (PEDOT:PSS), a widely used conductive polymer for emerging soft electronics, was employed as a model fluid in this study. A sub‐1 µL‐volume drop was gently dispensed onto the glass substrate using a precision pipette in the chamber, and its evaporation behavior was recorded using a high‐speed imaging technique (**Figure** **1**a). Under vacuum conditions, the rate of drop evaporation was notably accelerated due to the significant difference between the vapor pressure at the drop's surface and the ambient pressure within the vacuum chamber (Figure 1b). The chamber pressure (*P*_c_) varied between 101 and 0.67 kPa, and the target pressures were reached within 20 s (Figure 1c). The humidity monitored inside the chamber was also reduced rapidly down to 3–21%, depending on the final pressure (Figure 1d). Decreasing partial pressure of water molecules in the chamber increases the partial pressure difference across the air–liquid interface of the sessile drop. The lifetime of a drop (*t*_f_), defined as the duration until completion of evaporation, was dramatically reduced from 606 to 49 s by lowering *P*_c._ This significant decrease indicates that a reduced pressure environment substantially accelerates the evaporation rate, as shown in Figure 1e. Additionally, the relationship between the water boiling point and ambient pressure is detailed in Figure 1f, showing that the water‐based solution can transition from the liquid phase to the gas phase at room temperature when the chamber pressure reaches approximately 2 kPa.

**Figure 2:**
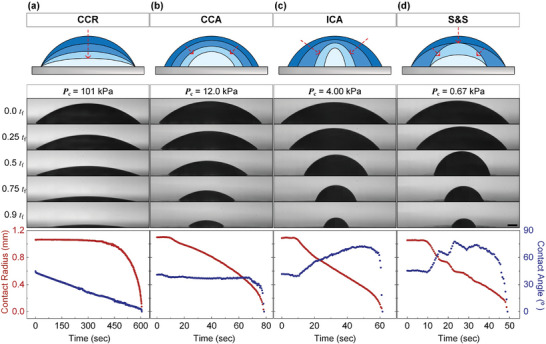
a–d) Visualizations of the side view of a sessile drop and the evolution of both the contact radius and angle characterize the evaporation mode of a sessile drop at each pressure in 101, 12.0, 4.00, and 0.67 kPa (see Video S1, Supporting Information). The sequence image shows the pressure conditions representing four evaporation modes; Constant Contact Radius (CCR) mode, Constant Contact Angle (CCA) mode, Increasing Contact Angle (ICA) mode, Stick and Slip (S&S) mode. The scale bar is 200 µm.

High‐speed imaging has unveiled, for the first time, that the evaporation of the sessile drop under vacuum conditions progresses through four distinct modes: Constant Contact Radius (CCR), Constant Contact Angle (CCA), Increasing Contact Angle (ICA), and Stick and Slip (S&S) modes (**Figure** **2**, see also Video S1, Supporting Information). Throughout the evaporation process, changes in contact radius and angle were quantified with 0.5‐s intervals to capture the dynamics of the drop. Under atmospheric conditions, evaporation predominantly follows a CCR mode, with the contact angle decreasing continuously until the final stages of evaporation.^[^ ^36^ ^]^ However, after undergoing transition regimes at 34.7 and 20.0 kPa, a further reduction in *P*_c_ down to 12 kPa induces a complete shift from the CCR to the CCA in the evaporation mode, with the contact angle stabilizing at ≈30°.^[^ ^37^ ^]^ The dynamics of the contact angle exhibit significant changes across varying pressure conditions. While the contact angle decreases consistently under atmospheric pressure and stabilizes at 30° at 12 kPa, it begins to increase as pressure further reduces down to 4 kPa, nearing its vapor pressure. This novel behavior of an evaporating drop – increasing contact angle while the contact radius decreases – has not been previously observed. Finally, when the pressure falls below 1 kPa, at which the water‐based fluid exceeds its vapor pressure at room temperature, a cyclic stick‐slip motion dominates the evaporation process, resulting in a distinct pattern of sudden movements interspersed with static phases.^[^ ^38^, ^39^ ^]^

**Figure 3:**
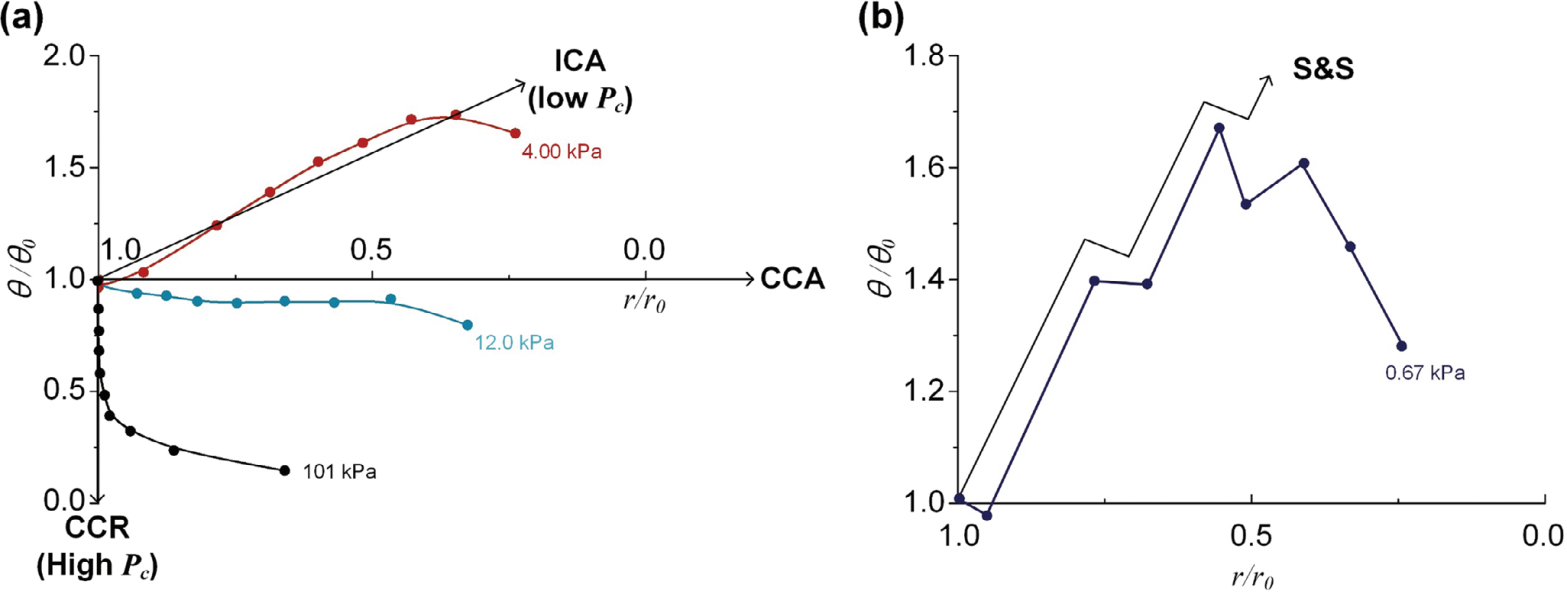
a) *θ*(r) diagram of three drops with different evaporation modes at 101, 12.0, and 4.00 kPa. b) *θ*(r) trajectories of S&S mode at 0.67 kPa. Each data point is recorded with a spacing at 0.1 volume ratio intervals.

To analyze different evaporation modes, we reconstruct the *θ*(*r*) trajectories using normalized contact radius and contact angle for each mode – CCR, CCA, ICA, and S&S as shown in **Figure** **3**. These trajectories, recorded at 0.1 volume ratio intervals, provide comprehensive insights into evaporation modes. In the ideal CCR mode, the *θ*(*r*) trajectory follows a vertical line at *r*/*r_0_* = 1, while in the ideal CCA mode, it maintains a horizontal line at *θ*/*θ_0_* = 1.^[^ ^23^ ^]^ Typically, evaporation occurs within a bounded rectangle between CCR and CCA modes. At atmospheric pressure, the evaporation follows the CCR trajectory, shifting toward the CCA line at 12.0 kPa, and further reduction to 4.00 kPa induces depinning of the contact line and a rapid increase in contact angle, with the *θ*(*r*) trajectories aligning with the ICA (Figure 3a). The evaporation at 0.67 kPa, which is below the vapor pressure at room temperature, leads to discrete, stepwise movements of contact line (Figure 3b). The lower pressure below 12.0 kPa enhances the contact line mobility, resulting in specific *θ*(*r*) trajectories that preserve the geometrical shape of the drop. The vacuum evaporation contributes to increasing the mobility of contact lines.

**Figure 4:**
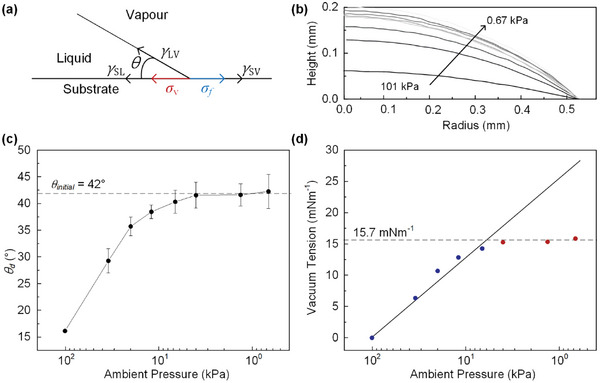
a) Schematics of force balance at contact line during vacuum evaporation. b) The contour of a sessile drop at the contact line when depinned at different pressures. c) The depinned contact angle according to the ambient pressure. The grey dot line is the average initial contact angle. d) Calculated vacuum tension at each pressure using modified Young's equation. The grey dop line shows vacuum tension when the contact line is depinned at the average initial contact angle. The surface tension of PEDOT:PSS and the substrate are 72.5 and 44.7 mN∙m^−1^, respectively.

The primary distinction in drop evaporation between atmospheric and low‐pressure conditions lies in the behavior of the depinning contact line, which is determined by the equilibrium of forces at the contact line. The force balance can be expressed using the modified Young‐Laplace equation (**Figure** **4**a).^[^ ^40^ ^]^

(1) $$\gamma_{LV}\cos\theta_{d}+\gamma_{SL}-\;\left(\gamma_{SV}+\sigma_{f}\right)=0\;\mathrm{at}\;\mathrm{atmospheric}\;\mathrm{pressure}$$

where *γ*_LV_, *γ*_SL_, and *γ*_SV_ denote the liquid‐vapor surface tensions, the solid‐liquid surface tensions, and the solid‐vapor surface tensions, respectively, and *θ*_d_ is the dynamic contact angle. The term *σ*_f_, often referred to as frictional tension due to surface heterogeneity, approaches zero at equilibrium.^[^ ^41^ ^]^ For drop evaporation at atmospheric pressure, given that the contact angle constantly diminishes while maintaining a constant contact radius, the term *γ*_LV_cos*θ* is subject to increase. Consequently, to maintain the triple line equilibrium as represented by the modified Young‐Laplace equation, *σ*_f_ must increase. The process of drop depinning initiates when *θ*_d_, the depinning contact angle, reaches 16.3° at a pressure of 101 kPa. Subsequently, with a reduction in *P*_c_ to 6.67 kPa during which *θ*_d_ increases to its initial equilibrium value of 42°. The *θ*_d_ remains saturated and does not change at lower *P_c_*. Figure 4b,c display the drop contours at the contact line and their corresponding contact angles upon depinning at various pressures.

With the changes in *θ*_d_ upon depinning at vacuum conditions, it becomes evident that the traditional Young‐Laplace equation does not adequately describe a force balance of the interfacial stresses under vacuum conditions. This leads to the following modification.

(2) $$\gamma_{LV}\cos\theta_{d}+\gamma_{SL}+\sigma_{v}-\;\left(\gamma_{SV}+\sigma_{f}\right)=0\;\mathrm{at}\;\mathrm{vacuum}\;\mathrm{conditions}$$

where *σ_v_* represents the additional force term due to vacuum pressure, the so‐called vacuum tension. We measured the surface energy of the substrate and the surface tension of the model fluid to be 44.7 and 72.8 mNm^−1^, respectively. The variable *σ_v_* with pressures was obtained by subtracting the Equation (2) from (1). Figure 4d illustrates that *σ_v_* escalates from zero and exhibits an increase with pressure reductions up to ≈15 kPa.

**Figure 5:**
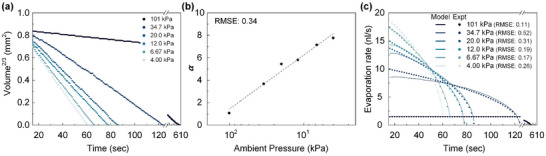
a) Volume^2/3^ variation over the time of evaporating a sessile drop under pressure higher than the vapor pressure of the water. The volume is calculated from the contact radius and angle, assuming the shape of the sessile drop is a spherical cap. b) Pressure factor α as a function of ambient pressure in diffusion‐limited evaporation model. c) The comparison of the evaporation rate between the experiments and modelling results based on a modified diffusion‐limited evaporation equation.

We next aimed to assess the validity of the widely used diffusion‐limited evaporation model for sessile drops that evaporate under subatmospheric pressures in a vacuum environment. In diffusion‐limited evaporation, the kinetics of drop evaporation is controlled by the rate at which vapor molecules can escape from the liquid's surface and diffuse through the surrounding air. The theory suggests that for a spherical sessile drop, the mass loss rate due to evaporation should be proportional to its surface area and thus independent of its volume.^[^ ^42^ ^]^ Under reduced ambient pressures, the evaporation is characterized by a linear decay of the drop volume to the two‐thirds power (*V* ^2/3^) over time (**Figure** **5**a). The drop evaporates more quickly as ambient pressure is reduced, which aligns with the expectation that lower pressures would lead to increased evaporation rates due to reduced air density around the drop, enhancing the vapor concentration gradient that drives the diffusion process. This observation indicates a surface area‐dependent evaporation process of the diffusion‐limited model.

To theoretically quantify the vacuum evaporation process, we modified the traditional diffusion‐limited evaporation model^[^ ^43^ ^]^ by adopting an ambient pressure factor α as

(3) $$-\frac{dV}{dt}\left(r,\theta \right)=\alpha \frac{\pi r\left(t\right)Dc_{s}}{\rho }\left(1-RH\right)g\left(\theta \left(t\right)\right)$$

where *r* is the contact radius, *D* is the diffusion coefficient of vapor in the air, *ρ* is the density of the fluid, *c*_s_ is the vapor concentration at the liquid‐vapor interface, *RH* is the relative humidity in the chamber and the function g(*θ*)^[^ ^44^ ^]^ is given by

(4) $$g\left(\theta \right)=\tan\left(\frac{\theta }{2}\right)+8\int_{0}^{\infty }\frac{\cosh^{2}\left(\theta \tau \right)}{\sinh\left(2\pi \tau \right)}\;\tanh\left[\tau \left(\pi -\theta \right)\right]d\tau$$

where *θ* is the contact angle.

The pressure factor is the ratio of the experimental volume *V* to the theoretic volume *V*_T_, which is obtained by integrating the diffusion‐limited evaporation equation over time, given by

(5) $$\alpha =\frac{V}{V_{T}}$$

(6) $$V_{T}=\int_{0}^{t_{f}}\frac{\pi r\left(t\right)Dc_{s}}{\rho }\left(1-RH\right)g\left(\theta \left(t\right)\right)dt)$$

The pressure factor is sensitive to changes in ambient pressure, showing a consistent decrease as the pressure is reduced (Figure 5b). Figure 5c compares experimental data and our theoretical model predictions of evaporation rates for different pressures. Under atmospheric conditions, a drop exhibits a constant evaporation rate, which is consistent with the CCR evaporation mode. In contrast, a reduction in ambient pressure results in a continuous decrease in the evaporation rate over the drop's lifetime, indicating that evaporation accelerates as the pressure lowers. Across all pressures, the suggested theoretical model agrees with the overall trend of the experimental evaporation rates, demonstrating its reliability in predicting drop behavior under these varied conditions.

**Figure 6:**
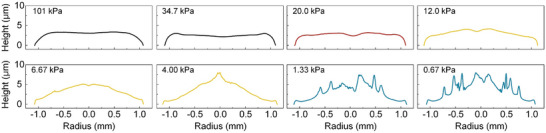
The film profiles of PEDOT:PSS in eight pressures after evaporation as measured by stylus profilometry. The deposition pattern is changed from a coffee ring to a mountain shape as the ambient pressure decreases. Under the vapor pressure of the solvent, the multi‐ring pattern emerges.

The evolution of evaporation modes under varying ambient pressures significantly influences the resulting deposition patterns of PEDOT:PSS polymers on the substrate, as illustrated in **Figure** **6**. Initially, the CCR mode evaporation in an atmospheric environment migrates the polymer chains toward the pinned contact line driven by capillary flow, resulting in weak coffee ring formation. The vacuum environment promotes the solvent's evaporation at the contact line, effectively overcoming the resistance offered by weak capillary flow and facilitating the transition to the CCA at 12.0 kPa. Reduced pressure causes the vapor‐liquid interface to contract, which actively gathers polymers and directs them toward the central area. Particularly in the ICA mode, where a pressure decreases to 6.67 kPa and further to 4 kPa, the polymers are notably transported toward the drop's apex and develop a dense central deposit. An increasing contact angle and a constantly decreasing contact radius enhance polymer delivery to the center, creating a concentrated region at the core of the final deposit. At the lowest pressure, S&S mode leads to a markedly nonuniform pattern, characterized by several deposition lines. This low‐pressure environment causes the water‐based ink to exceed its boiling point at room temperature, which induces complex flow motion in the near edge region, which is also found in the evaporation of a drop on a heated substrate.^[^ ^45^, ^46^ ^]^ Notably, at the transition pressure that marks the mode shift from CCR to CCA, a simultaneous decrease in contact radius and angle results in a uniformly distributed final deposit.^[^ ^22^ ^]^ It is particularly noteworthy that this uniform morphology of the dried drop was achieved through environmental control alone, without modifying the fluid or surface properties.

## 3 Conclusion

This study has significantly advanced our understanding of the evaporation dynamics of sessile drops containing conducting polymer under varying pressure conditions, particularly in a vacuum environment. Our high‐speed imaging investigations captured the transition through distinct evaporation modes – CCR, CCA, ICA, and S&S – each distinctly influenced by changes in vacuum levels only without altering the fluid or substrate characteristics. In particular, the ICA mode, occurring near vapor pressure, has not been observed in drop dynamics under atmospheric conditions, highlighting a unique aspect of evaporation that appears specifically under reduced‐pressure environments. These modes provide a comprehensive understanding of how environmental conditions directly impact drop evaporation mechanisms and the resultant film morphology. We observed that key factors such as the dynamics of the depinning contact line and variations in contact angle play a critical role in shaping the evaporation process, with reduced ambient pressures significantly accelerating evaporation rates. The adaptation of a modified diffusion‐limited evaporation model incorporating an ambient pressure factor effectively captures the evolution of droplet volume under these conditions, aligning well with our empirical data and underscoring its utility in predicting drop behavior across a spectrum of pressures.

Our research demonstrates the potential of manipulating vacuum conditions to significantly enhance film uniformity and quality, making it particularly valuable for industrial applications such as electronics manufacturing and printing processes. Unlike traditional film morphology tuning methods, which often rely on altering substrate or liquid properties or adjusting temperature under atmospheric conditions, our approach allows for a substantial reduction in evaporation time without the need to modify material properties to achieve the desired film morphology. This versatility makes it applicable to a wide range of polymers and substrate conditions (Figures S1–S3, Supporting Information). We believe the precise control of the vacuum environment would offer the most effective strategy for both reducing manufacturing time and improving the performance of practical electronic devices.

## 4 Experimental Section

### Material Preparation

PEDOT:PSS 1.0 wt.% (Heraeus Clevios PH 1000, Germany) ink dispersed in water was chosen as the polymers to deposit a thin film of evaporated drop on a glass substrate. The glass slides (20 × 25 mm) were used as a base substrate for depositing the polymer inks. The contact angles of water and diiodemethane on the glass substrate were 65.32° and 53.28°, respectively. The surface energy of the glass substrate calculated by the Owens, Wendt, Rabel, and Kaelble (OWRK) method was 44.7 mNm^−1^. All substrates were cleaned before use by placing the substrate in an ultrasonic bath of de‐ionized water, acetone, and IPA for 10 min, respectively. The remaining liquid on the substrate was removed by baking the substrates at 100 °C.

### Vacuum Evaporation High‐Speed Imaging

The vacuum chamber, measuring 20 cm in length, width, and height, was constructed from anodized aluminum, with quartz glass windows embedded in the side walls. The connection line to a vacuum pump was incorporated to remove the air in the chamber. The pressure in the chamber was regulated by adjusting the valve integrated into the connection line. The pressure gauge (InstruTech CVM201 Super Bee Convection, USA) in chamber was electrically connected to a data acquisition board (National Instrument NI‐6353, USA), and the value of pressure and relative humidity was recorded over time by humidity sensor (Econarae ETH‐01D, Korea). Side view images were taken using a high‐speed camera (Photron Fastcam SA‐3, USA) and a high‐magnification zoom lens with a working distance of 83 mm (Navitar 12X Zoom lens, USA). The sequence of evaporation images was recorded at a frame rate of 60 frames per second. The camera and vacuum pump were operated simultaneously when the equipment received a trigger pulse from DAQ. The drop was formed pendant drop from the pipet and was gently placed on the glass substrate at room temperature. To analyze the droplet during evaporation, its contour was extracted from the obtained images at 0.5‐s intervals using an image processing algorithm created by LabVIEW program. The contact radius and contact angle were then measured from the extracted droplet contour. The volume of drop was calculated based on the contact radius and angle assuming that the sessile drop was a spherical cap. The camera and pump operations, as well as the image processing software employed in this experiment, were developed in‐house using LabVIEW program. The film profiles were measured by using a stylus profilometer (Bruker DektakXT, USA).

## Conflict of Interest

The authors declare no conflict of interest.

## Supporting information



Supporting Information

Supplemental Video 1

## Data Availability

The data that support the findings of this study are available from the corresponding author upon reasonable request.
