# Supplementary material for: Evaporative Morphology Tuning of Conducting Polymer Films Under Controlled Vacuum Conditions
Source: Adv Sci (Weinh). 2024 Dec 23;12(6):2408790. doi: 10.1002/advs.202408790 (PMC11809338; doi:10.1002/advs.202408790)
Supplement: Supplementary file 1 — Supporting Information [file ADVS-12-2408790-s002.docx]

Supporting Information

Evaporative Morphology Tuning of Conducting Polymer Films under Controlled Vacuum Conditions

*Seongju Kim, Byung Mook Weon, Dong Jin Kang, and Sungjune Jung^*^*


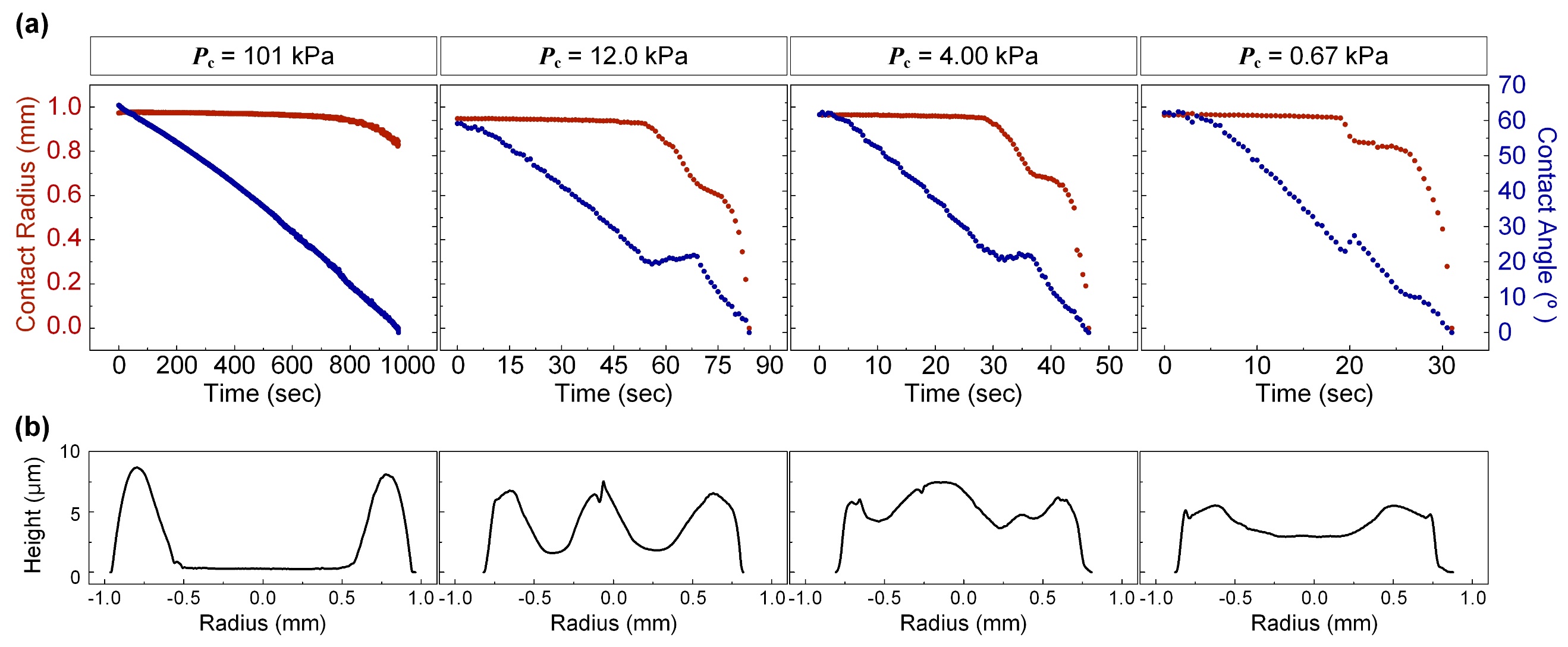


**Figure S1.** (a) The evaporation dynamics of polyvinlpyrrolidone (PVP) drop at 101, 12.0, 4.00 and 0.67 kPa. (b) Film profiles of PVP at 101, 12.0, 4.00 and 0.67 kPa.

The polymer ink was prepared by dissolving 1 wt% polyvinylpyrrolidone (PVP) in water and stirred overnight to ensure complete polymer dispersion. A sub-1 μL drop of the ink was gently deposited onto a glass substrate and allowed to evaporate at varying pressures. At atmospheric pressure, the evaporation dynamics followed the constant contact radius (CCR) mode, resulting in the coffee-ring effect. However, as the pressure was reduced to 12.0 kPa, the evaporation dynamics changed, causing polymer accumulation at the center of the drop. Further pressure reduction enhanced the central accumulation, indicating that the decrease in pressure suppresses the coffee-ring effect.


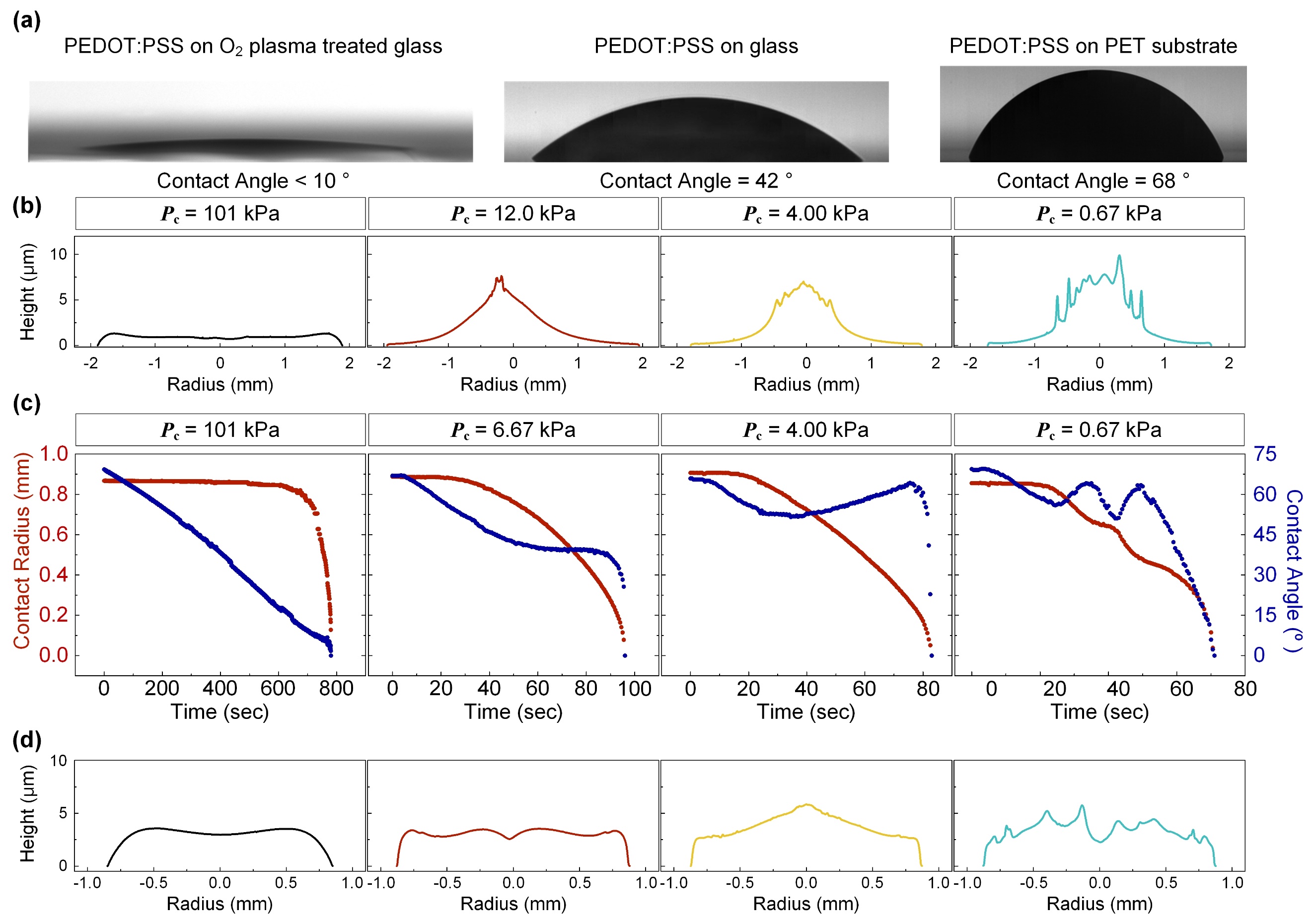


**Figure S2.** (a) The contact angles depending on the substrate: O_2_ plasma treated glass, the glass and PET substrate. (b) The film profiles of PEDOT:PSS O_2_ plasma treated glass on at 101, 12.0, 4.00 and 0.67 kPa. (c) The evolution of the contact angle and radius of PEDOT:PSS under the pressures: 101, 6.67, 4.00 and 0.67 kPa. (d) The film profiles of PEDOT:PSS on PET substrate at 101, 6.67, 4.00 and 0.67 kPa.

PEDOT:PSS ink exhibits a small contact angle on oxygen plasma-treated substrates, making it difficult to accurately extract the contact radius and angle from side-view images. The diameter of the PEDOT:PSS drop exceeds the capillary length of 3 mm, preventing the drop from being modeled as a spherical cap. To investigate the effect of hydrophilic substrate on the film formation during vacuum evaporation, we compared the film profiles of PEDOT:PSS on the oxygen plasma-treated glass under different ambient pressures. At 101 kPa, the film on the plasma-treated glass is thinner and has a larger diameter compared to untreated glass, with a weak coffee-ring effect. As the pressure decreases, there is a significant accumulation of polymer at the center. At 0.67 kPa, the film profile also reveals multi-ring patterns. To further explore the effect of different wettability, we investigated the evaporation dynamics of PEDOT:PSS ink on a polyethylene terephthalate (PET) substrate with a contact angle of 68°. The evaporation behavior of PEDOT:PSS ink on PET under ambient pressure was similar to that observed on glass, and the film profiles showed comparable trends in pressure-dependent transitions. These findings suggest that our method can be extended to other types of substrates.


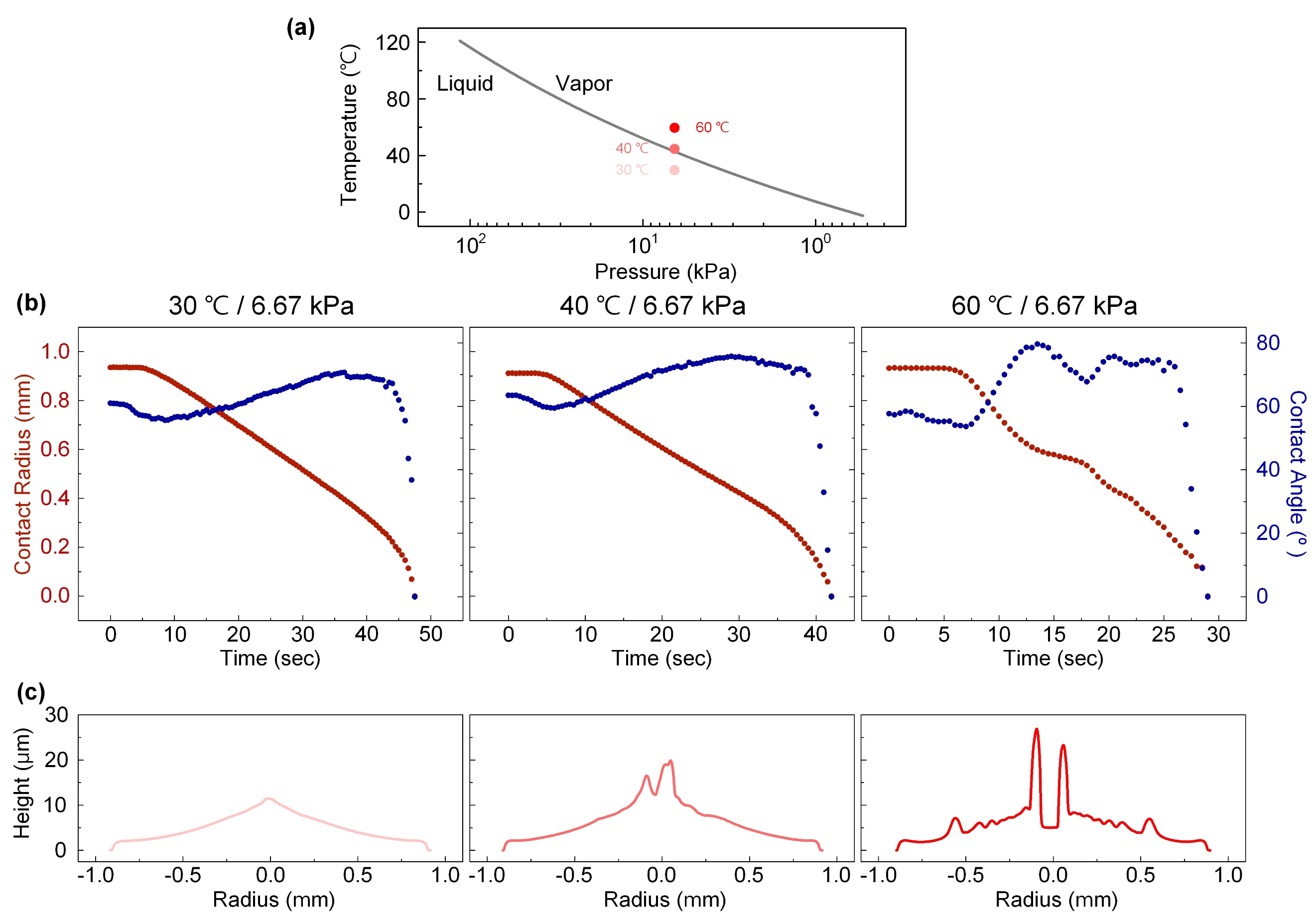


**Figure S3.** (a) The evaporation conditions on the graph of the dependence of the water boiling point on pressure. (b) The evolution of the contact angle and radius under 6.67 kPa at different temperatures: 30, 40 and 60 ℃. (c) The film profiles under 6.67 kPa at different temperatures: 30, 40 and 60 ℃.

We examined the effect of different temperatures—30°C, 40°C, and 60°C—on the evaporation dynamics under a pressure of 6.67 kPa, where the boiling point of water is 38°C. At 30°C, which is below the boiling point, the evaporation process followed the ICA mode, leading to the formation of a mountain-shaped film profile due to the slow evaporation rate below the boiling point. At 40°C, close to the boiling point, the drop also evaporated in the ICA mode but reached a higher maximum contact angle, resulting in a similarly shaped film profile with an increasing peak at the center. At 60°C, which exceeds the boiling point of water at 6.67 kPa, the evaporation occurred in the S&S mode, similar to evaporation dynamics seen at 0.67 kPa at room temperature. This transition to the S&S mode produced film profiles that exhibited distinctive multi-ring patterns, indicating rapid evaporation and uneven polymer deposition. These results exhibit that temperature also plays a critical role in influencing both the evaporation mode and the resulting film morphology.
